# A clinical study to assess the influence of acupuncture at “Wang’s Jiaji” acupoints on limb spasticity in patients in convalescent stage of ischemic stroke: study protocol for a randomized controlled trial

**DOI:** 10.1186/s13063-019-3464-7

**Published:** 2019-07-10

**Authors:** Huanqin Li, Dehuai Long, Bin Li, Huilin Liu, Tingting Ma, Tingying Wu, Martin Eriksson, Yali Wen, Jia Wei, Wei You, Yinxia Liu, Xiaobai Xu, Yajie Zhang, Linpeng Wang, Jingqing Sun

**Affiliations:** 1 Acupuncture and Moxibustion Department, Beijing Hospital of Traditional Chinese Medicine, Capital Medical University, No.23 Meishuguanhou Street, Dongcheng District, Beijing, 100010 China; 2 Beijing key Laboratory of Acupuncture Neuromodulation, Beijing, China; 3 Capital Medical University, Beijing, China; 4 Beijing University of Chinese Medicine, Beijing, China

**Keywords:** Ischemic stroke, Acupuncture, Limb spasticity, “Wang’s Jiaji” acupoints, Convalescent stage, Clinical study

## Abstract

**Background:**

Stroke is characterized by high morbidity, high mortality, and high disability. Spasticity, one of the most common complications after stroke, may reduce the potential success of rehabilitation and has a detrimental effect on stroke patients’ daily function and quality of life. Moreover, the long-term management of spasticity is a financial burden to patients and increases societal costs. The current treatments, mainly including physical therapy, oral drugs, drug injection therapy, and surgical interventions, have been used to reduce spasticity. However, every conventional approach has its limitations. Acupuncture at the “Wang’s Jiaji” acupoints, based on the experience of the famous old doctor of traditional Chinese medicine (TCM) Le Ting Wang in treating post-stroke limb spasm, has been widely practiced in our department. This intervention has effectively avoided the controversy around acupuncture at local acupoints on the limbs, and is easy to apply without side effects. Our previous studies had found that acupuncture at the “Wang’s Jiaji-points” can reduce the occurrence and severity of spasticity occurring after stroke in the early stage (the first 21 days). In this study, we chose patients in the convalescent stage, 1–6 months after stroke, so as to study the efficacy and the specific intervention time of “Wang’s jiaji” in the convalescent stage after stroke.

**Methods:**

This is a randomized, controlled, and single-blind study. Patients in the convalescent stage within 1–6 months of ischemic stroke will be selected as subjects. A total of 100 subjects will be randomly assigned to two groups. The acupuncture group will be given acupuncture treatment five times a week; the medicine group will be given 10mg baclofen three times a day. These two groups will continue to receive current usual care for the prevention and treatment of cerebrovascular diseases, but drugs that affect muscle tone will not be allowed. The treatment will last for 2 weeks. The primary outcome measurement is the simplified Fugl-Meyer Assessment. The secondary outcome measurements are the Modified Ashworth Scale, Modified Barthel Scale, and the H-reflex, F response, and H/M ratios of electromyography. All outcome measurements are assessed at baseline, 2 weeks, 4 weeks, and 12 weeks after first treatment except the electromyography, which is assessed at baseline and 2 weeks after first acupuncture.

**Discussion:**

This trial aims to evaluate the effects and the specific intervention time of “Wang’s Jiaji” acupoints on spasticity after stroke.

**Trial registration:**

ISRCTN registry, ISRCTN31511176. Registered on 29 August 2017.

Version number of protocol 2016-2-1161

Version date of protocol: 2016-1

**Electronic supplementary material:**

The online version of this article (10.1186/s13063-019-3464-7) contains supplementary material, which is available to authorized users.

## Background

**Fig. 1:**
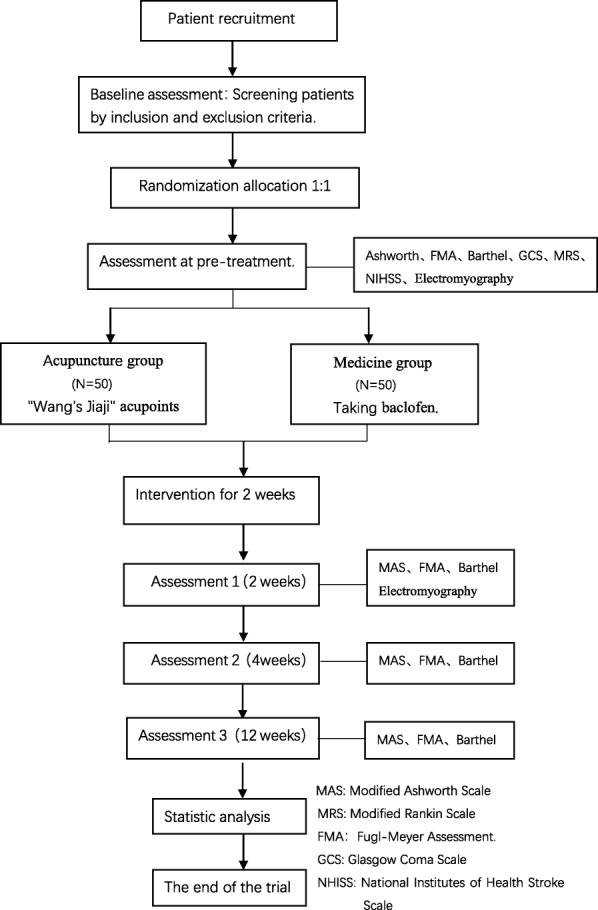
Trial design. ITT intention to treat

Stroke is characterized by high levels of morbidity, mortality, and disability [1]. The two major types of stroke are ischemic and hemorrhagic, the former accounting for 80% and the latter about 20% of strokes [2]. Spasticity is the most common post-stroke complication [3]. Morbidity in post-stroke spasticity is about 65% worldwide and is 80% to ∼ 90% in China [3, 4]. Spasticity may reduce the potential success of rehabilitation [5] and can also have a detrimental effect on stroke patients’ quality of life and daily function [6]. Moreover, the long-term management of spasticity is a financial burden to patients and increases societal costs [1]. Different treatments, such as physical therapy, oral drugs, drug injection therapy, and surgical interventions, have been used to reduce spasticity. However, every conventional approach has its limitations [7]. For physical therapy, there is no consensus about the application standard and there is no randomized controlled study to confirm the efficacy [8]. Oral drugs have potential adverse effects, such as sedation, respiratory depression, fatigue, nausea and so on. Drug injection and surgical treatment are aimed at patients with severe spasticity, and require a high level of skill in their application. In addition, drug and surgical treatments are high-cost interventions with high risk and limited effects. Therefore, their clinical application is somewhat restricted [9]. In recent years, acupuncture therapy has become one of the most important methods to improve dysfunction after stroke [10] and has been shown to reduce spasticity with minimal side effects [11, 12]. Acupuncture at “Wang’s jiaji” acupoints, based on the experience of the famous old doctor of traditional Chinese medicine (TCM) Le Ting Wang, in treating post-stroke limb spasticity in our department, avoids the controversy around acupuncture at local acupoints of the limbs, and is easy to apply without side effects. Wang’s jiaji acupoints include 16 points that are located 0.3 cun (about 1 cm) lateral to the lower border of the 2nd, 4th, 6th, 8th, 10th, and 12th thoracic vertebrae, and the 2nd and 4th lumbar vertebrae. Our previous studies have shown that acupuncture at Wang’s jiaji acupoints can prevent spasticity or reduce its severity. However, subjects in these studies were exclusively convalescent patients recruited within the first 21 days after stroke [13]. The rate of post-stroke spasticity has been reported to be 4–27% during the first 6 weeks after onset, 19% at 3 months, and 21.7–42.6% at 4 and 6 months [14]. Therefore, we chose patients in the convalescent stage 1–6 months after stroke as the subjects of our study. Baclofen, approved by the Food and Drug Agency (FDA), is a widely used anti-spasticity drug [15]. So we use baclofen as a comparator, in order to study the efficacy of Wang’s jiaji acupoints, the time points of intervention, and the possibility of reducing the severity of spasticity occurring in the convalescent stage of 1–6 months after stroke (Fig. 1; Additional file 1).

## Methods

### Population

A target sample of 100 participants will be recruited from acupuncture wards in Beijing Hospital of Traditional Chinese Medicine affiliated to Capital Medical University. The trial will take place from January 2017 to December 2018.

### Sample size

According to the literature, “xiuqiong Chen, chuyu Yang. To observe the curative effect of combining rehabilitation training with oral baclofen on post-stroke spasticity”. After the treatment of combining oral baclofen with rehabilitation training about 3–6 months, the FMA score of subjects is from 29. 91±7. 63 increased to 58.18 ± 8.32. In our previous work, the FMA score is from 26.68±13.50  increased to 52.32 ± 24.60 after treatment with acupuncture at “Wang’s Jiaji” acupoints. If the FMA score in the proposed study is increased by 28.27 points or more in the acupuncture group, this will indicate clinical significance. If the FMA score is 10 points higher in the acupuncture group than in the oral baclofen group, with α = 0.05 and test efficiency 1−β = 0.90, we will require at least 42 subjects in each group. Allowing for subjects lost to follow-up and subjects withdrawn from the study (below 20%), we will need at least 50 subjects in each group, hence a total sample size of at least 100 subjects.

### Recruitment of participants

Recruiting is conducted at both the inpatient and outpatient departments by physicians who are all trained to the same level. Patients who are potentially eligible for the study will be well-informed about the content of the study, including the free oral medicine, full care of disease, and the researcher’s contact information. The screening form will then be completed via a face-to-face interview with a researcher and assessment prior to the randomization.

### Inclusion criteria

The inclusion criteria are:

1. Diagnosis of ischemic stroke, in accordance with the diagnostic criteria for cerebral infarct (including atherosclerotic thrombotic cerebral infarct, cerebral embolism, lacunar infarct).
2. Onset within 1–6 months (≥1 month and ≤ 6 months).
3. Age 40–80 years.
4. National Institute of Health stroke scale (NIHSS) scores ≥ 4 and ≦ 21 points.
5. Modified Ashworth Scale (MAS) grade ≥ 2 and ≤ 4.
6. Glasgow Coma Scale (GCS) scores ≥ 7 points, and fully conscious.
7. First stroke or without severe disability from a previous stroke: modified Rankin Scale (mRS) score ≤ 3 points.
8. Ischemic cerebral infarction confirmed by computed tomography (CT) or magnetic resonance imaging (MRI) of the head.
9. Agreement to participate in this trial and sign the informed consent form.

### Exclusion criteria

The exclusion criteria are:

1. Limb dystonia caused by brain or other diseases.
2. Participation in other trials within the last 3 months.
3. Serious primary heart, liver, kidney, or hematopoietic system diseases or psychiatric illness.
4. Taking drugs other baclofen to improve muscular tension (or taking baclofen within the previous 3 days)
5. Allergy to baclofen.
6. Pregnant or lactating women.
7. Congenital disabilities.

### Randomization and blinding

Participants will be randomly assigned to the acupuncture group or the oral medicine group following the randomization principle, which employs block randomization to generate the random allocation sequence using SPSS software and prepares predetermined computer-generated, opaque, sealed, randomization envelopes. The number of participants allocated to the acupuncture group and the oral medicine group is at a ratio of 1:1. Opaque envelopes labeled with patient study number will be opened to allocate participants into the predefined group. Allocation concealment will not be broken until the completion of the study. The random allocation assignment will be performed only when the potential subject is confirmed to be eligible for this study and the written informed consent has been obtained.

### Interventions

#### Acupuncture group

Wang’s jiaji acupoints include the 16 points located 0.3 cun (about 1 cm) lateral to the lower border of the 2nd, 4th, 6th, 8th, 10th, and 12th thoracic and the 2nd and 4th lumbar vertebrae.

The procedure is as follows: the patients are required to maintain the lateral decubitus position, with the hemiplegic limbs uppermost. Piercing vertically, needles are inserted 10–25 mm in depth and manually manipulated by lifting, thrusting, and rotating methods with uniform reinforcing-reducing techniques to produce the sensation known as “deqi”. For patients with aphasia, deqi will be defined as the feeling of heaviness under the practitioner’s fingers; for patients without aphasia, deqi will be defined as the patient experiencing a sensation of soreness, numbness, or swelling.

The acupuncture technique involves mild reinforcing and attenuating, retaining the needle in the acupoints for 30 min. All the needles inserted in the acupoints need to be in line horizontally and vertically. Treatment will be given once a day, five times a week for 2 weeks.

#### Medicine group

Participants will take baclofen 10 mg three times per day for 2 weeks.

#### Basic treatment

All patients in the acupuncture and medicine groups will continue to receive current usual care for the prevention and treatment of cerebrovascular diseases, but drugs that affect muscle tone will not be allowed.

### Primary outcome measures

The simplified Fugl-Meyer scale is used to evaluate the upper and lower limb motor function on the affected side at baseline, 2 weeks, 4 weeks, and 12 weeks after the treatment with acupuncture or medicine.

### Secondary outcome measures

The secondary outcome measures are:

1. The MAS, to evaluate muscular tension at baseline, 2 weeks, 4 weeks and 12 weeks after the first treatment with acupuncture or medicine.
2. The MBS, to evaluate patients’ activities of daily living at baseline, 2 weeks, 4 weeks, and 12 weeks after the first treatment with acupuncture or medicine.
3. Electromyography, to evaluate the level of spasticity at baseline and 2 weeks after the first treatment with acupuncture or medicine.

### Withdrawal and dropout

Participation in the study will be terminated if at any stage the participant refuses to continue, withdraws consent, violates the recruiting criteria, or fails to completes the 2-week treatment. The trial will be stopped if the principle investigator believes that there are unacceptable risks of serious adverse events.

### Adverse events

Adverse events, such as abnormal changes on laboratory test results and expected adverse reactions (including hematoma, needle sickness, pneumothorax, etc.), will be reported. All related and unexpected adverse events will be recorded in detail on the case report form. Participants with mild and moderate adverse events will be treated for their symptoms and closely monitored by the researcher. Severe adverse events will be reported to the Research Ethics Committee, which will provide medical advice to the research team within 48 h and determine whether the participant is suitable to continue the study.

### Data management

Study procedures and documents will be monitored by Beijing Clinical Research Quality Promotion and Control Center. The trial will involve specialized data management personnel and institutions, so personal information on the participants will be kept strictly confidential throughout the study period and thereafter. Scale evaluators and investigators are responsible for data management. Scale evaluators who have received specialized rehabilitation training are especially responsible for the evaluation of the simplified Fugl-Meyer scale, MAS, and MBS. Other scales and the case report forms are recorded by the investigator. Data obtained during the study will be confidentially treated and will be stored in a locked filing cabinet. All data will be processed using participants’ ID codes, which will not be linked to participants’ personal information. The datasets will not be shared with anyone outside the collaborative group without the express permission of each collaborator. The investigator is the only person who can modify the data. If a mistake is found in the original data, the investigator should underline the original data, enter the modified data next to it, then sign and date it. The data will be managed in accordance with the Data Protection Act of 1998. If there is any doubt about the data, the original record of the data may be checked.

### Statistical analysis

The collection of study data and statistical analysis will be performed by an assigned expert. The major outcome target will be statistically analyzed mainly using the intention-to-treat (ITT) method and the per-protocol (PP) method. The secondary outcome target will mainly be analyzed PP. All statistical tests will be two-sided. A *P* value less than or equal to 0.05 will be considered statistically significant. Measurement data will be described by the mean ± standard deviation. Comparison between two groups is based on the analysis of variance or non-parametric test. The paired *t* test or non-parametric test will be adopted to compare values to the baseline value. Enumeration data will be described by the number and percentage of participants. The two groups will be compared using the chi-square test, the Fisher exact probability method, or non-parametric test.

## Discussion

We have presented the design and the protocol for a randomized controlled trial of acupuncture at Wang’s jiaji acupoints compared to treatment with baclofen. Completion of this trial will help to explore the curative effect and intervention time of acupuncture at the Wang’s Jiaji acupoints in treating limb spasticity in patients in the convalescent stage after ischemic stroke.

Acupuncture at the Wang’s jiaji acupoints according to the famous old doctor of TCM Le Ting Wang’s 60 years of clinical experience has been widely employed in our department in treating post-stroke spasticity. It is easy to apply with minimal side effects and can avoid controversy around stimulating the limbs. Our previous studies had shown that acupuncture at Wang’s jiaji acupoints can reduce the occurrence and severity of spasticity after stroke in the early stage (the first 21 days) [13]. In this study, we will recruit patients with limb spasticity in the convalescent stage, to study the efficacy and intervention time of acupuncture at Wang’s jiaji. Baclofen was chosen as the oral drug for the control group because it is safe, popular, and effective [15]. Based on the scale evaluation in previous studies, we will also include several electrophysiological measures as part of the outcome assessment, such as H- reflection, F-wave, and the H max/M max ratio, in order to provide quantitative information about spasticity [18]. In recent years, the key role of electromyography in the evaluation of spasticity, especially in post-stroke spasticity, has been confirmed [16–18]. According to experience in our previous clinical practice, we consider that in this study, the efficacy of acupuncture at Wang’s Jiaji acupoints in improving limb spasticity in the convalescent stage of ischemic stroke would be equal to or better than oral baclofen, and the curative effect on patients at 1 or 2 months after stroke would better reflect an advantage in the early stage of convalescence treatment. According to previous research, in patients with spasticity, the hyperexcitability of the stretch reflex is neurophysiologically characterized by shorter latencies and greater amplitudes of the H-reflex [18, 19], an increase in F-wave amplitude [20], and an increase in the H max/M max ratio [21]. The application of electrophysiological measures is expected to reduce the interference of subjective factors with scale evaluation. According to modern neurophysiology, the posterior and anterior rami of the spinal nerve and the sympathetic trunk are distributed in the region of Wang’s Jiaji acupoints, with each of the posterior rami of the spinal nerve connected by fiber with the adjacent 1–2 upper and lower posterior rami nerves. Needling at Wang’s Jiaji acupoints may decrease its severity by affecting the interactions of the spinal cord and motor neurons, adjusting the spinal stretch reflex, and balancing the function of the motion system that adjusts the state of limb muscle tension.

## Trial status

This trial is currently recruiting participants.

## Additional file

Additional file 1: The Standard Protocol Items: Recommendation for Interventional Trials (SPIRIT) checklist for this trial. (DOCX 25 kb)

## Data Availability

Not applicable.

## Abbreviations

CT: Computed tomography
FDA: Food and Drug Administration
FMA: Fugl-Meyer Assessment
GCS: Glasgow Coma Scale
ITT: Intention-to-treat
MAS: Modified Ashworth Scale
MBS: Modified Barthel Index Scale
MRI: Magnetic resonance imaging
MRS: Modified Rankin Scale
NHISS: National Institutes of Health Stroke Scale
PP: Per-protocol
SPSS: Statistical Package for the Social Sciences
TCM: Traditional Chinese medicine
